# Deficiency of the lipoprotein lipase pathway modifies the phenotypic expression of genetically determined lipoprotein(a)

**DOI:** 10.1016/j.jlr.2026.101164

**Published:** 2026-10-05

**Authors:** Sotirios Tsimikas, Xiaohong Yang, Elaine Waldman, Fan Lyu, Veronica J. Alexander, Santica M. Marcovina

**Affiliations:** 1 Vascular Medicine Program, Division of Cardiovascular Medicine, University of California San Diego, La Jolla, CA, USA; 2 Ionis Pharmaceuticals, Carlsbad, CA, USA; 3 Medpace Reference Laboratories, Cincinnati, OH, USA

**Keywords:** lipoprotein(a), familial chylomicronemia syndrome, lipoprotein lipase, apolipoprotein(a) isoforms, oxidized phospholipids, triglyceride-rich lipoproteins, lipoprotein metabolism, apolipoprotein B

## Abstract

Plasma lipoprotein(a) [Lp(a)] concentrations are primarily genetically determined, but whether disruption of triglyceride-rich lipoprotein metabolism modifies Lp(a) expression is unknown. Familial chylomicronemia syndrome (FCS) provides a human model to investigate this relationship. In the phase 3 Balance trial, 66 participants with genetically confirmed FCS (triglycerides ≥880 mg/dl) underwent Lp(a) measurement, apo(a) isoform phenotyping, and measurement of oxidized phospholipid (OxPL)-apo(a) and OxPL-apolipoprotein B (apoB). Results were compared with 66 age- and sex-matched participants with moderate hypertriglyceridemia from Essence-TIMI 73b. Median Lp(a) concentrations were similar (9.77 [4.64–33.6] versus 11.8 [5.23–90.4] nmol/L; *P* = 0.148), but the Lp(a) distribution in FCS was shifted downward, driven by fewer individuals with high Lp(a) concentrations despite similar apo(a) isoform distributions. The inverse relationship between apo(a) isoform size and Lp(a) was preserved (P for trend <0.001), with lower Lp(a) in FCS after stratification by apo(a) isoform size (*P* = 0.006). Median (IQR) Lp(a) was lower in FCS with <17 kringle IV (KIV) repeats (67.4 [49.2–93.7] versus175 [145–324] nmol/L; *P* = 0.018) and 17–20 KIV repeats (23.9 [9.37–55.3] versus138 [17.1–192] nmol/L; *P* = 0.012). OxPL-apo(a) and OxPL-apoB were lower in FCS (*P* = 0.026 and *P* < 0.0001, respectively). The OxPL-apo(a)/OxPL-apoB ratio was approximately 70% lower (0.60 [0.27–1.69] versus2.05 [1.49–2.79]; *P* < 0.0001) and showed a pronounced inverse trend across apo(a) isoform size in FCS (*P* < 0.001), consistent with altered relative OxPL carriage among apoB-containing lipoproteins. Olezarsen had no consistent effects on Lp(a) or OxPL biomarkers. These findings demonstrate that functional deficiency of the LPL pathway modifies the phenotypic expression of genetically determined Lp(a) and alters OxPL distribution among apoB-containing lipoproteins.

Elevated lipoprotein(a) [Lp(a)] is an independent and likely causal risk factor for atherosclerotic cardiovascular disease and calcific aortic valve disease (1). Circulating Lp(a) concentrations vary more than 1000-fold among individuals and are largely determined by the number of kringle IV (KIV) repeats within the *LPA* gene, with smaller apo(a) isoforms generally associated with higher Lp(a) levels (2). Although Lp(a) concentrations are primarily genetically determined, metabolic factors may also influence Lp(a) production, assembly, and clearance 3, 4. Population studies have consistently reported weak inverse associations between triglyceride and Lp(a) concentrations 5, 6, 7, 8, but whether severe disturbances in triglyceride-rich lipoprotein metabolism modify the genetically determined relationship between apo(a) isoform size and plasma Lp(a) remains unknown.

Familial chylomicronemia syndrome (FCS) represents the most extreme human disorder of triglyceride-rich lipoprotein metabolism and therefore provides a unique human model of disrupted triglyceride-rich lipoprotein metabolism to examine whether severe metabolic perturbation can modify the genetically determined Lp(a) phenotype. (9) Extreme chylomicronemia may alter formation or stability of Lp(a), which requires covalent attachment of apo(a) to an LDL-like particle. Although reduced Lp(a) concentrations have previously been reported in lipoprotein lipase deficiency, apo(a) isoform characteristics and oxidation-specific biomarkers were not evaluated (10). Accordingly, whether severe hypertriglyceridemia alters the relationship between apo(a) isoform size, plasma Lp(a), and OxPL remains unknown.

Lp(a) is the principal lipoprotein carrier of oxidized phospholipids (OxPLs) in human plasma and circulating OxPL-apo(a) and OxPL-apolipoprotein B (apoB) closely parallel Lp(a) concentrations across a wide range of Lp(a) levels 11, 12, 13. However, it is unknown whether this relationship is preserved in metabolic states characterized by profoundly reduced Lp(a). If Lp(a), the principal plasma carrier of OxPL, is reduced, OxPL may likewise decline or redistribute to non-Lp(a) apoB-containing lipoproteins. Higher OxPL-apoB levels have been previously noted in subjects with small apo(a) isoforms in the Dallas Heart Study across 3 racial/ethnic populations (12). Determining how OxPL is distributed in this setting may provide new insights into the metabolic interplay between triglyceride-rich lipoproteins and Lp(a).

Because *APOC3* inhibition with olezarsen is now an established treatment for FCS and is incorporated into contemporary guidelines (14), we also explored whether marked triglyceride lowering modifies circulating Lp(a) and OxPL biomarkers in this unique population. The phase 3 Balance trial (15) provided a unique opportunity to characterize plasma Lp(a), apo(a) isoform expression, and OxPL distribution in genetically confirmed FCS. We hypothesized that severe disruption of triglyceride-rich lipoprotein metabolism modifies the phenotypic expression of circulating Lp(a) across apo(a) isoform sizes, producing a downward shift in the apo(a) isoform–Lp(a) relationship and altering relative OxPL carriage among apoB-containing lipoproteins.

## Materials and methods

### Study design and populations

This is a post hoc substudy of the Balance (ISIS 678354-CS3; NCT03335355) trial, a phase 3, randomized, double-blind, placebo-controlled study evaluating the efficacy and safety of olezarsen, an antisense oligonucleotide targeting *APOC3* messenger ribonucleic acid, in 66 adults with genetically confirmed FCS (15). Eligible individuals were ≥18 years of age, had fasting triglyceride concentrations ≥880 mg/dl (≥∼10 mmol/L), and documented loss-of-function variants in *LPL*, *APOC2*, *APOA5*, *GPIHBP1*, or *LMF1*. Exclusion criteria included recent pancreatitis (<4 weeks). Participants were effectively randomized 1:1:1 to receive subcutaneous olezarsen 50 mg, olezarsen 80 mg, or placebo once monthly for 12 months, followed by observational follow-up for 13 weeks. The protocol was approved by the institutional review board at each participating center, and the trial was performed in accordance with the guidelines of the International Council for Harmonisation and the principles of the Declaration of Helsinki. All the participants provided written informed consent before enrollment.

To determine whether the Lp(a) phenotype observed in FCS differed from that seen in less severe hypertriglyceridemia, an external comparator cohort was selected from the Essence-TIMI 73b CT angiography substudy, in which baseline Lp(a), OxPL-apo(a), and OxPL-apoB measurements were available. Essence-TIMI 73b participants were frequency matched to Balance participants by age within 5 years and sex 16, 17.

### Lp(a) quantification

Lp(a) was quantified using the Roche Tina-quant Gen.2 immunoturbidimetric assay (Medpace Reference Laboratories), which served as the primary assay for both the Balance and Essence-TIMI 73b cohorts. To assess the robustness of the findings across analytical methods, Balance samples were also measured using the UCSD monoclonal antibody-based, isoform-independent Lp(a) assay (18). Both assays are calibrated to the WHO/IFCC SRM-2B reference material, and results are reported in nmol/L. All samples were analyzed in duplicate, with intra-assay and inter-assay coefficients of variation <5%. Longitudinal Lp(a) concentrations in the Balance cohort were measured at baseline and on study days 85, 169, 253, and 365, and at baseline in the Essence-TIMI 73b substudy.

### Apo(a) isoform analysis

Apo(a) isoform size was determined at Medpace Reference Laboratories by a highly sensitive SDS-agarose gel electrophoresis method calibrated with 5 plasma samples with a defined number of KIV repeats determined by pulsed-field gel electrophoresis on the DNA of the same donors as the plasma samples 19, 20. Isoform size was reported as the number of KIV repeats per allele. When two isoforms were detected, relative band intensities were quantified to calculate the percent contribution of each isoform to total apo(a) signal.

### OxPL and immune biomarker measurements

OxPL content was measured using validated ELISAs for OxPL-apo(a) and OxPL-apoB (UCSD), employing the E06 monoclonal antibody specific for phosphocholine-containing OxPL epitopes (11). OxPL analyses used available samples from 66 Balance and 52 Essence-TIMI 73b participants.

To assess whether any observed patterns in OxPL biomarkers reflect generalized systemic oxidative stress, independent of specific lipoprotein carriage, in Balance we also measured IgG and IgM autoantibodies to a malondialdehyde (MDA) mimotope, a peptide surrogate for MDA-modified epitopes commonly used as a marker of broader oxidative modification using microtiter plates coated with an MDA-peptide mimotope as described previously (21), at baseline and on study days 85, 169, 253, and 365.

### Lipid and apolipoprotein measurements

Total cholesterol concentration in serum samples was determined using Beckman Coulter reagent and calibrator on a Beckman Coulter AU5800 instrument. Based on results obtained from quality control (QC) samples with low, medium, and high cholesterol concentrations, intra-assay precision (coefficient of variation) ranged from 0.6% to 1.5%, and inter-assay precision ranged from 0.7% to 3.9%.

Triglyceride concentration in serum samples was determined using Beckman Coulter reagent and calibrator on a Beckman Coulter AU5800 instrument. Based on QC samples with low, medium, and high triglyceride concentrations, intra-assay precision ranged from 0.4% to 1.5%, and inter-assay precision ranged from 0.6% to 3.7%.

HDL-C concentration was determined using Beckman Coulter reagent and calibrator on a Beckman Coulter AU5800 instrument. Cholesterol in HDL was measured in serum supernatants obtained after precipitation of apoB-containing lipoprotein particles with dextran sulfate. Based on QC samples with low, medium, and high HDL-C concentrations, intra-assay precision ranged from 0.1% to 4.8%, and inter-assay precision ranged from 1.0% to 6.7%.

LDL-C concentration was determined by the beta-quantification procedure after separation of lipoprotein particles by preparative ultracentrifugation. Based on QC samples with low, medium, and high LDL-C concentrations, intra-assay precision ranged from 0.4% to 4.2%, and inter-assay precision ranged from 1.0% to 3.6%. Accuracy of all lipid analyses was verified regularly by comparison with results obtained using the Abell-Kendall reference method.

apoB concentration in serum samples was determined by an immunonephelometric method using Siemens reagent and calibrator on a Siemens BN II System. Based on QC samples with low, medium, and high apoB concentrations, intra-assay precision ranged from 1.1% to 2.2%, and inter-assay precision ranged from 2.6% to 3.5%.

ApoB-48 concentration was determined using an apoB-48 ELISA kit (FUJIFILM) on a Tecan Sunrise instrument. Intra-assay precision determined using QC samples ranged from 3.8% to 8.9%, and inter-assay precision ranged from 8.6% to 14.6%. The same analytical methods were used for variables included in the Balance and Essence-TIMI 73b comparisons.

### Statistical analysis

Continuous variables are presented as median (interquartile range [IQR]) unless otherwise indicated. Mean (SD) values are provided for selected descriptive and graphical summaries.

Between-cohort comparisons were performed using two-sided Wilcoxon rank-sum tests. Within-category comparisons were performed using two-sided Wilcoxon rank-sum tests and are reported as nominal *P* values to describe the pattern of differences within the four predefined apo(a) isoform strata. The overall cohort comparison across isoform strata was assessed using the van Elteren test, with isoform category (<17, 17–20, 21–24, and >24 KIV repeats) as the stratification factor. Ordered trends across isoform categories were assessed separately within each cohort using the two-sided asymptotic Jonckheere–Terpstra test. Within-category comparisons reported in Table 2 used two-sided Wilcoxon rank-sum tests and were not adjusted for multiple comparisons.

**Table 2:** Comparison of Lp(a) concentrations by predominant apo(a) isoform size in the Balance FCS and ESSENCE moderate hypertriglyceridemia cohort

Predominant apo(a) Isoform Size, KIV Repeats	Essence, n	Essence Lp(a), Nmol/L, Median (IQR)	Balance, n	Balance Lp(a), Nmol/L, Median (IQR)	Lower Median Lp(a) in FCS, %	Nominal *P*
<17	5	175 (145–324)	7	67.4 (49.2–93.7)	61	0.018
17–20	16	138 (17.1–192)	17	23.9 (9.37–55.3)	83	0.012
21–24	14	24.5 (4.20–51.7)	10	10.3 (4.60–20.2)	58	0.464
>24	31	7.30 (3.70–12.2)	28	6.26 (3.90–10.6)	14	0.534

Predominant apo(a) isoform size was defined by the apo(a) isoform with the greater relative expression. Lp(a) concentrations were measured using the Roche Tina-quant Lipoprotein(a) Gen.2 assay in both cohorts. Percent lower was calculated as 100 × (Essence median − Balance median)/Essence median. Within-category comparisons between cohorts were performed using two-sided Wilcoxon rank-sum tests because Lp(a) concentrations were right-skewed. KIV kringle IV; Lp(a), lipoprotein(a). Within-stratum *P* values are nominal and are provided descriptively. The overall Balance-versus-Essence comparison across apo(a) isoform strata was assessed using the van Elteren test (*P* = 0.006).

Associations among Lp(a), OxPL biomarkers, MDA mimotope autoantibodies, and lipid variables were assessed using Spearman rank correlation coefficients. Differences in IgG and IgM MDA mimotope autoantibody levels across predominant apo(a) isoform categories were evaluated using the Kruskal–Wallis test. Analyses used available cases; observations with missing isoform-category or biomarker data were excluded from the relevant analysis.

All statistical analyses were performed using SAS version 9.4 (SAS Institute, Cary, North Carolina). All tests were two-sided, and *P* < 0.05 was considered statistically significant. Asymptotic *P* values used the SAS default continuity correction.

## Results

### Study population

Participants in Balance had the expected biochemical profile of FCS and differed from the age- and sex-matched Essence-TIMI 73b cohort (Table 1). Median fasting triglyceride concentrations were approximately nine-fold higher (2,335 [IQR 1,744–3,337] versus268 [201–359] mg/dl), whereas LDL-C measured by ultracentrifugation (17.5 [12.0–25.0] versus83.0 [66.5–117.0] mg/dl) and total apoB (62.4 [48.5–70.4] versus96.0 [82.2–118.0] mg/dl) were substantially lower. ApoB-48 concentrations were higher (9.40 [5.81–18.81] versus2.53 [1.43–4.30] mg/dl), consistent with severe impairment of triglyceride-rich lipoprotein metabolism.

**Table 1:** Summary of baseline laboratory characteristics in the balance and essence cohorts

Variable	Balance (N = 66)	ESSENCE (N = 66)	*P*-value
Age, yr	Matched	Matched	Matched
Sex, % female	Matched	Matched	Matched
Fasting triglycerides, mg/dL	2,335 (1,744–3,337)	268 (201–359)	<0.0001
LDL-C (ultracentrifugation)	17.5 (12.0–25.0)	83.0 (66.5–117.0)	<0.0001
Total apoB, mg/dL	62.4 (48.5–70.4)	96.0 (82.2–118.0)	<0.0001
Total apoB, nmol/L	1,217 (944-1,371)	1,871 (1,601–2,300)	<0.0001
ApoB-48, mg/dL	9.40 (5.81–18.81)	2.53 (1.43–4.30)	<0.0001
Total cholesterol, mg/dL	274 (212–378)	177 (154–219)	<0.0001
OxPL-apo(a)^c^	3.09 (0.98–14.63)	29.9 (4.94–81.8)	0.0260
OxPL-apoB^c^	4.93 (3.83–7.10)	8.93 (3.68–35.3)	<0.0001
OxPL-apo(a)/OxPL-apoB ratio	0.60 (0.27–1.69)	2.05 (1.49–2.79)	<0.0001
Lp(a), nmol/L			
Median (IQR)	9.77 (4.64–33.6)	11.8 (5.23–90.4)	0.1480
Mean (SD)	23.8 (31.8)	65.8 (103.5)	
Range	2.26–169.5	5.30–438.6	
Predominant apo(a) isoform^a^, #KIV repeats	23 (18–29)	24 (19–29)	0.5811
Minor apo(a) isoform^b^, #KIV repeats	25 (21–27)	24 (23–29)	0.4112
Predominant apo(a) isoform, Expression, %^a^	92 (78–100)	93 (82–100)	0.4003
Minor apo(a) isoform, Expression, %^b^	8 (0–22)	7 (0–18)	0.4003

Data are presented as median (interquartile range) unless otherwise indicated.

*P*-values for continuous variables were calculated using a two-sided Wilcoxon rank-sum test based on the normal approximation.

a Predominant apo(a) isoform was defined as the isoform with the greater relative expression. All participants with an evaluable apo(a) result had a detectable predominant apo(a) isoform.

b Minor apo(a) isoform analyses were restricted to participants with two detectable apo(a) isoforms. Participants with only one detectable apo(a) isoform were excluded from analyses of minor apo(a) isoform size and relative expression because a second isoform could not be reliably quantified.

c 66 participants in Balance and 52 participants in ESSENCE had available measurements.

Median (IQR) Lp(a) concentrations were similar in Balance and Essence-TIMI 73b (9.77 [4.64–33.6] versus11.8 [5.23–90.4] nmol/L; *P* = 0.148) but mean (SD) Lp(a) concentrations were substantially lower in Balance (23.8 [31.8] versus 65.8 [103.5] nmol/L), reflecting fewer individuals with high Lp(a) concentrations (Table 1). Median (IQR) OxPL-apo(a) concentrations were nearly 10-fold lower in Balance than Essence-TIMI 73b (3.09 [0.98–14.63] versus29.9 [4.94–81.8] nmol/L; *P* = 0.026), whereas OxPL-apoB concentrations were approximately 45% lower (4.93 [3.83–7.10] versus8.93 [3.68–35.3] nmol/L; *P* < 0.0001). Consequently, the OxPL-apo(a)/OxPL-apoB ratio was approximately 70% lower in Balance (0.60 [0.27–1.69] versus2.05 [1.49–2.79]; *P* < 0.0001), consistent with altered distribution of OxPL among non-Lp(a) apoB-containing lipoproteins. For comparison, previously reported median (IQR) values in patients undergoing coronary angiography were 3.8 (2.7–8.4) nmol/L for OxPL-apoB and 11.7 (4.5–37.0) nmol/L for OxPL-apo(a), with an OxPL-apo(a)/OxPL-apoB ratio of 3.00. (22).

Despite these marked differences in circulating Lp(a) and OxPL biomarkers, predominant and minor apo(a) isoform size distributions and relative isoform expression were similar between cohorts.

### Distribution of Lp(a) and OxPL-related biomarkers

The kernel density distributions of Lp(a), OxPL-apo(a), OxPL-apoB, and the OxPL-apo(a)/OxPL-apoB ratio are shown in Figure 1 , and their empirical cumulative distribution functions are shown in Figure 2. Compared with the age- and sex-matched Essence-TIMI 73b cohort, Balance participants had fewer individuals with high Lp(a) concentrations.

**Fig. 1:**
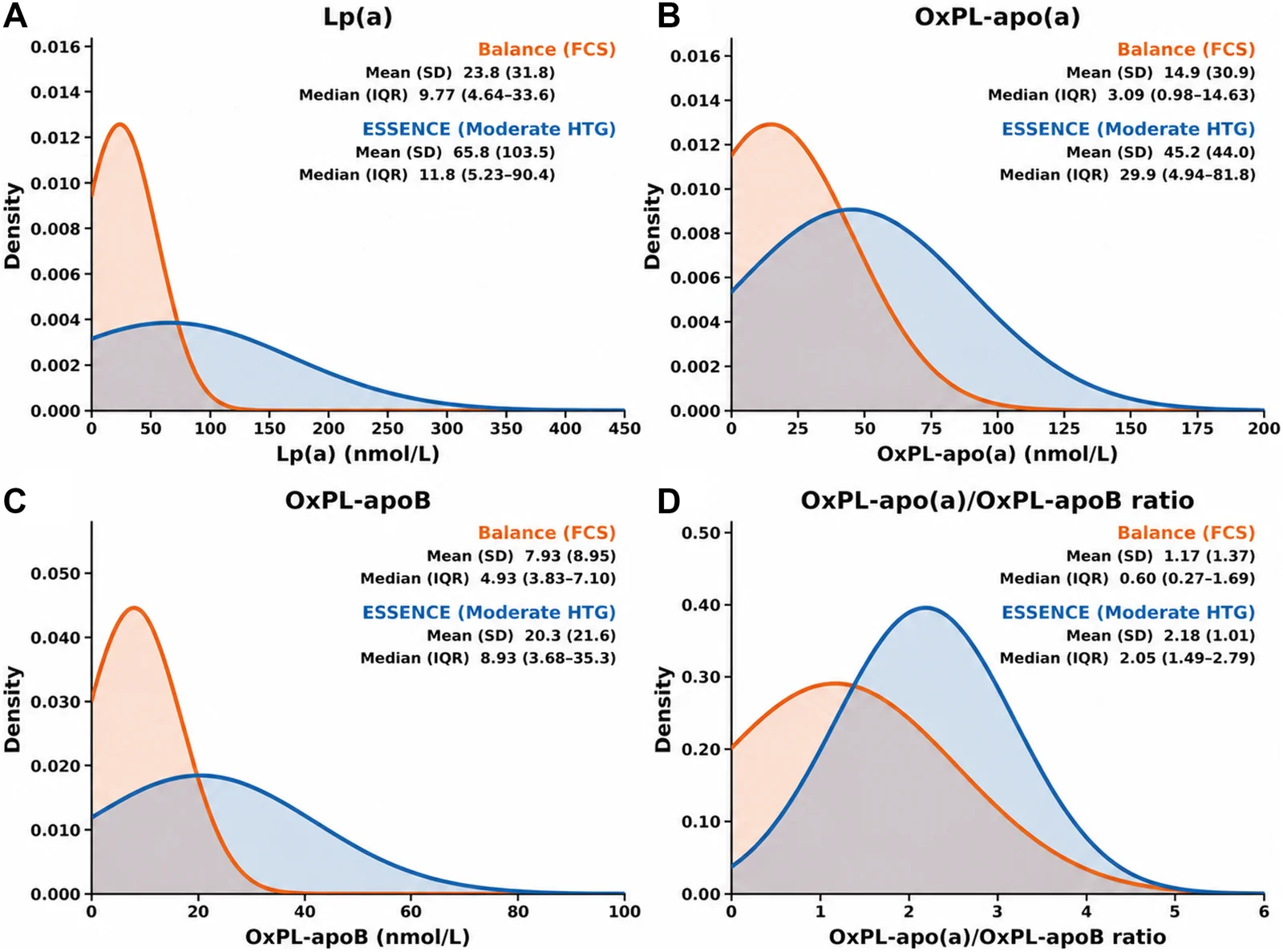
Distributions of Lp(a) and oxidation-specific biomarkers in the Balance (FCS) and Essence-TIMI 73b (moderate hypertriglyceridemia) cohorts. Kernel density plots of (A) Lp(a), (B) OxPL-apo(a), (C) OxPL-apoB, and (D) the OxPL-apo(a)/OxPL-apoB ratio in the Balance cohort (orange) and the age- and sex-matched Essence-TIMI 73b moderate hypertriglyceridemia cohort (blue). Shaded areas are kernel density estimates; summary statistics within each panel are mean (SD) and median (IQR). Lp(a), OxPL-apo(a), and OxPL-apoB are in nmol/L; the ratio is unitless. FCS, familial chylomicronemia syndrome; Lp(a), lipoprotein(a); OxPL, oxidized phospholipid.

**Fig. 2:**
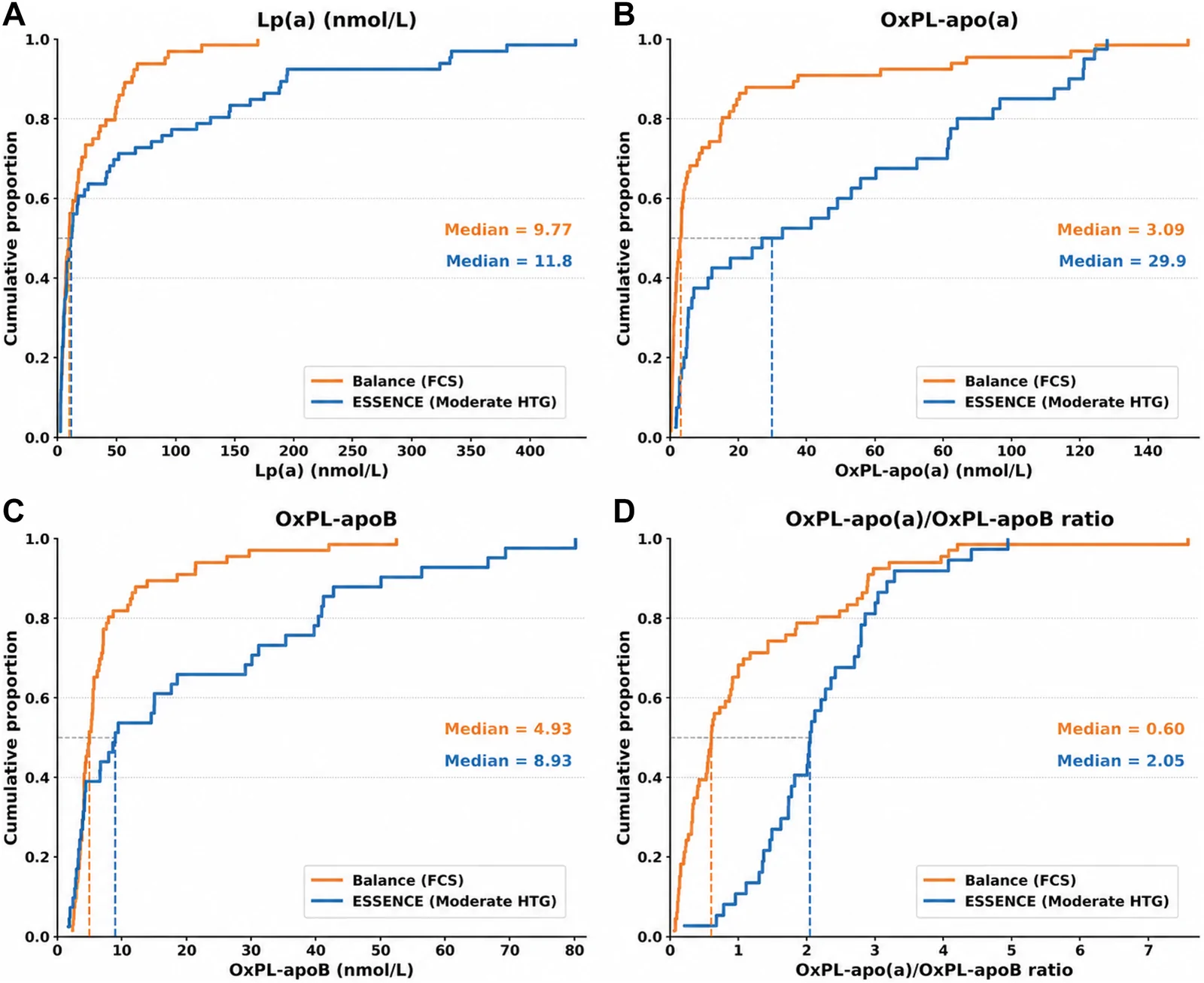
Empirical cumulative distribution functions of plasma Lp(a) and OxPL in participants in the Balance and Essence-TIMI 73b cohorts. Empirical cumulative distribution functions are shown for (A) Lp(a), (B) OxPL-apo(a), (C) OxPL-apoB, and (D) the OxPL-apo(a)/OxPL-apoB ratio in Balance cohort (orange) and the age- and sex-matched Essence-TIMI 73b moderate hypertriglyceridemia cohort (blue). For each curve, the y-axis indicates the cumulative proportion of participants with a value at or below the corresponding x-axis value. Dashed vertical lines indicate the cohort median, and median values are annotated within each panel. Lp(a), OxPL-apo(a), and OxPL-apoB are reported in nmol/L; the OxPL-apo(a)/OxPL-apoB ratio is unitless. The Balance distributions were shifted toward lower values relative to Essence-TIMI 73b, most prominently for OxPL-apo(a), OxPL-apoB, and the OxPL-apo(a)/OxPL-apoB ratio; for Lp(a), the difference was characterized primarily by fewer individuals with high concentrations. FCS, familial chylomicronemia syndrome; HTG, hypertriglyceridemia; Lp(a), lipoprotein(a); OxPL, oxidized phospholipids.

Measurement of Balance samples using the independent UCSD isoform-independent assay confirmed the reduced circulating Lp(a) concentrations in subjects with smaller isoforms. Mean ± SD Lp(a) concentration was 13.4 ± 28.6 nmol/L (median [IQR], 5.36 [2.24–14.4] nmol/L). Among the 64 participanants with measurements available by both methods, UCSD- and Roche-measured Lp(a) concentrations were strongly correlated (Spearman r = 0.81, *P* < 0.0001), supporting the robustness of the low Lp(a) phenotype across analytical methods.

Because apo(a) isoform size is the principal genetic determinant of circulating Lp(a), we next examined whether the marked reduction in Lp(a) observed in FCS could be explained by differences in apo(a) isoform size or relative isoform expression.

### Lp(a) concentrations are reduced within apo(a) isoform strata in FCS

Consistent with the established inverse relationship between apo(a) isoform size and circulating Lp(a), median Lp(a) concentrations decreased progressively with increasing predominant apo(a) isoform size in both cohorts (Table 2; P for ordered trend <0.001 in both cohorts). The overall Balance-versus-Essence-TIMI 73b comparison, stratified by predominant apo(a) isoform category, was significant (*P* = 0.006). The greatest separation was observed among participants with smaller apo(a) isoforms. For predominant isoform sizes <17 KIV repeats, median (IQR) Lp(a) was 67.4 (49.2–93.7) nmol/L in Balance versus 175 (145–324) nmol/L in Essence-TIMI 73b (*P* = 0.018). For isoform sizes 17–20 KIV repeats, median Lp(a) was 23.9 (9.37–55.3) versus 138 (17.1–192) nmol/L, respectively (*P* = 0.012). Differences were smaller among participants with 21–24 KIV repeats (10.3 [4.60–20.2] versus 24.5 [4.20–51.7] nmol/L) and >24 KIV repeats (6.26 [3.90–10.6] versus 7.30 [3.70–12.2] nmol/L). Similar findings were observed with the UCSD assay (supplemental Table S1).

Figure 3 further demonstrates that this downward displacement was evident in Balance with both the Roche and UCSD isoform-independent assays, supporting the robustness of the findings across analytical methods. The separation from Essence-TIMI 73b was greatest among participants with smaller apo(a) isoforms, whereas little difference was observed among those with the largest isoforms, indicating that the lower Lp(a) phenotype associated with FCS was most pronounced in individuals genetically predisposed to higher Lp(a) concentrations.

**Fig. 3:**
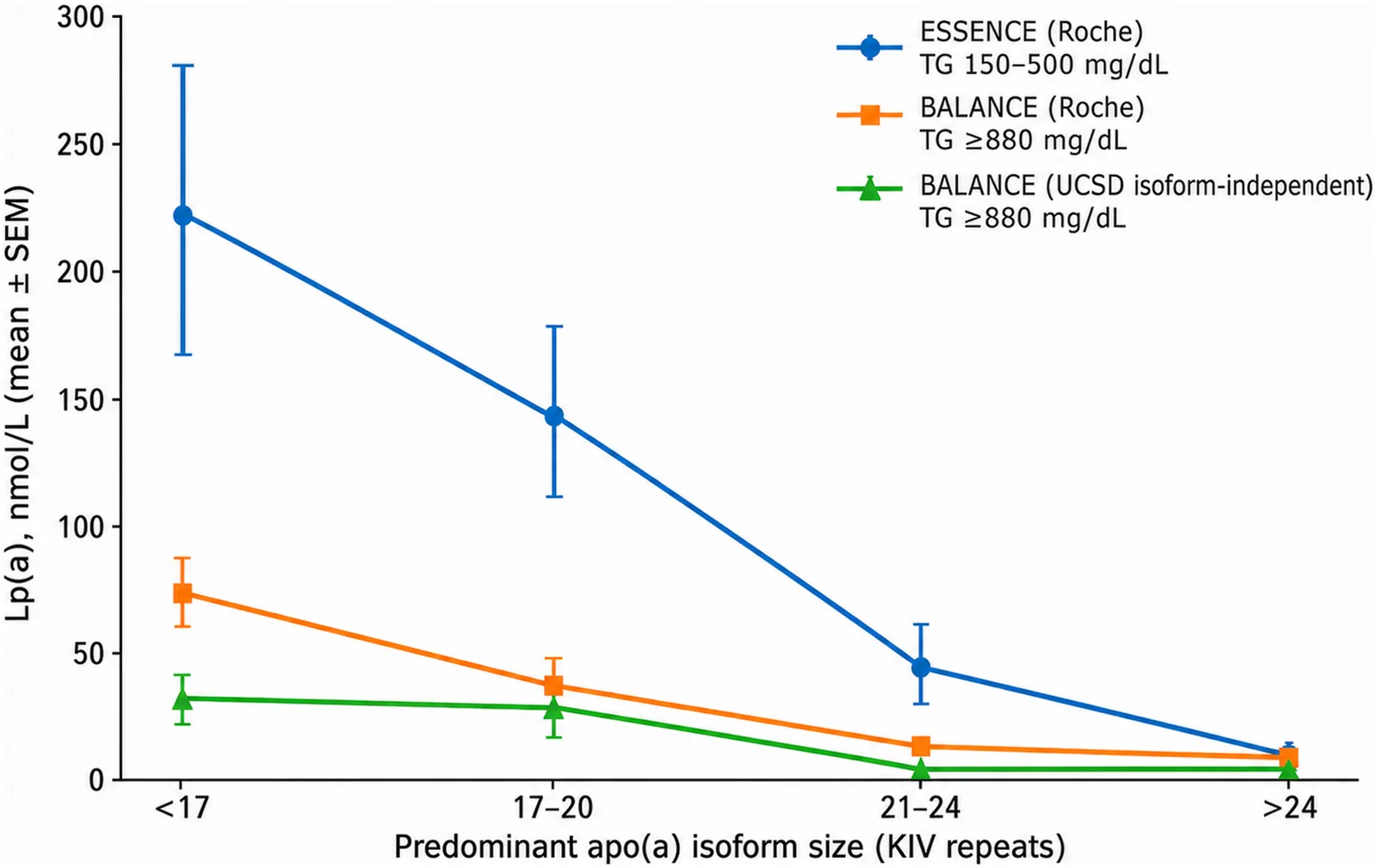
Plasma Lp(a) concentrations according to predominant apo(a) isoform size in the Balance and matched Essence-TIMI 73b cohorts. Mean (±SEM) plasma Lp(a) concentrations are plotted against predominant apo(a) isoform size (<17, 17–20, 21–24, and >24 kringle IV [KIV] repeats) for the Essence-TIMI 73b moderate hypertriglyceridemia cohort measured with the Roche assay (blue circles) and the Balance familial chylomicronemia syndrome cohort measured with both the Roche assay (orange squares) and the UCSD isoform-independent assay (green triangles). Error bars represent the standard error of the mean. In all series, plasma Lp(a) decreased with increasing apo(a) isoform size, preserving the expected inverse relationship between apo(a) isoform size and circulating Lp(a). Lp(a) concentrations were lower in Balance with both assays, with the greatest separation among participants with smaller apo(a) isoforms. Lp(a), lipoprotein(a); SEM, standard error of the mean; TG, triglycerides.

### Comparison of OxPL concentrations by predominant apo(a) isoform size in the Balance and Essence-TIMI 73b cohorts

OxPL-apo(a) declined progressively with increasing apo(a) isoform size in both cohorts (Table 3; P for ordered trend <0.001 in both cohorts), with an overall Balance-versus-Essence-TIMI 73b comparison of *P* < 0.001. OxPL-apoB also showed an ordered decline in Essence-TIMI 73b (*P* < 0.001) and Balance (*P* = 0.001), with an overall between-cohort *P* = 0.025. The OxPL-apo(a)/OxPL-apoB ratio showed a significant ordered trend in Essence-TIMI 73b (*P* = 0.031) and an inverse trend in Balance (*P* < 0.001); the overall Balance-versus-Essence-TIMI 73b comparison was *P* < 0.001 (Fig. 4).

**Table 3:** Comparison of OxPL concentrations by predominant apo(a) isoform size in the ESSENCE moderate hypertriglyceridemia cohort and the balance familial chylomicronemia syndrome cohort

Biomarker	Cohort	<17 KIV	17-20 KIV	21-24 KIV	>24 KIV	P for trend^a^	P Balance versus Essence^b^
OxPL-apo (a), nmol/L	Essence	84.2 (82.1–116.9)	83.5 (26.8–116.8)	47.7 (15.4–54.5)	4.92 (2.73–6.90)	<0.001	
	Balance	20.4 (14.9–61.7)	9.36 (2.86–22.2)	1.42 (1.03–3.62)	1.66 (0.73–3.66)	<0.001	<0.001
OxPL-apoB, nmol/L	Essence	40.3 (39.7–56.4)	33.2 (9.69–46.4)	12.2 (5.42–23.3)	3.57 (2.58–7.27)	<0.001	
	Balance	7.08 (4.15–21.4)	7.55 (4.34–13.8)	4.96 (3.26–6.42)	4.11 (3.57–5.45)	0.001	0.025
OxPL-apo (a)/OxPL-apoB ratio	Essence	2.12 (2.04–2.21)	2.38 (1.91–2.82)	3.17 (1.30–4.07)	1.49 (1.11–2.02)	0.031	
	Balance	2.89 (2.74–3.20)	1.17 (0.60–2.15)	0.44 (0.15–0.60)	0.42 (0.20–0.77)	<0.001	<0.001

Data are median (IQR), with IQR presented as Q1-Q3.

Available-case analysis: observations with missing Isoform 1 (KIV) or missing biomarker values are excluded.

a P for ordered trend was calculated using a two-sided asymptotic Jonckheere-Terpstra test across the ordered predominant apo(a) isoform categories (<17, 17–20, 21–24, and >24 KIV), separately within each cohort.

b P compares Balance with ESSENCE using a two-sided van Elteren stratified Wilcoxon rank-sum test, with Isoform 1 category (<17, 17–20, 21–24, and >24 KIV) as the stratification factor. Asymptotic *P* values were calculated using the SAS default continuity correction.

**Fig. 4:**
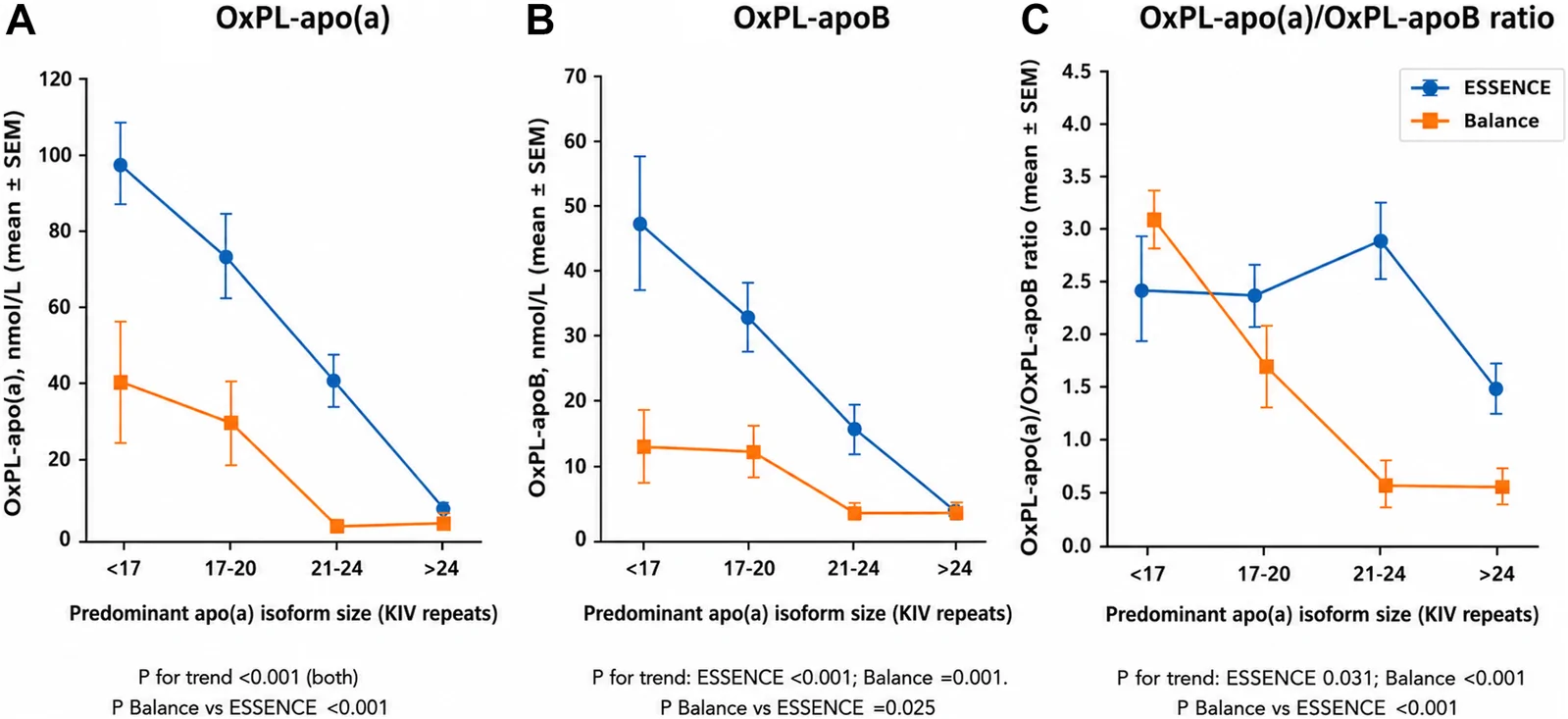
OxPL content according to predominant apo(a) isoform size in the Balance and Essence-TIMI 73b cohorts. Mean (±SEM) (A) OxPL-apo(a), (B) OxPL-apoB, and (C) the OxPL-apo(a)/OxPL-apoB ratio are shown by predominant apo(a) isoform category (<17, 17–20, 21–24, and >24 kringle IV [KIV] repeats) in the Essence-TIMI 73b moderate hypertriglyceridemia cohort (blue circles) and the Balance FCS cohort (orange squares). OxPL-apo(a) and OxPL-apoB showed an overall inverse relationship with increasing apo(a) isoform size in both cohorts, consistent with altered relative partitioning of OxPL away from Lp(a) among apoB-containing lipoproteins in FCS. The OxPL-apo(a)/OxPL-apoB ratio was relatively preserved across the three smaller isoform categories in Essence-TIMI 73b, whereas Balance showed a pronounced inverse trend with increasing isoform size, indicating a shift of OxPL toward non-Lp(a) apoB-containing lipoproteins in severe hypertriglyceridemia. Error bars represent the standard error of the mean. Lp(a), lipoprotein(a); OxPL, oxidized phospholipids; SEM, standard error of the mean.

IgG and IgM MDA mimotope autoantibody levels remained similar across apo(a) isoform categories (Kruskal–Wallis *P* = 0.287 and *P* = 0.290, respectively).

### Correlation analysis

Within the Balance cohort, circulating Lp(a) concentrations remained strongly associated with oxidation-specific biomarkers despite profound hypertriglyceridemia (supplemental Table S2). Lp(a) measured by the UCSD assay correlated closely with OxPL-apo(a) (Spearman *r* = 0.84, *P* < 0.0001) and OxPL-apoB (*r* = 0.63, *P* < 0.0001), with nearly identical relationships observed using the Roche assay (OxPL-apo(a): *r* = 0.81; OxPL-apoB: *r* = 0.55; both *P* < 0.0001). OxPL-apo(a) and OxPL-apoB were also strongly correlated with one another (*r* = 0.71, *P* < 0.0001).

### Correlations of Lp(a), OxPL-related biomarkers, and MDA autoantibodies with lipid variables

In the baseline Balance cohort, neither Lp(a) nor OxPL-related biomarkers showed meaningful correlations with triglycerides, apoC-III, LDL-C, HDL-C, apoB, apoB-48, or chylomicron triglycerides (supplemental Table S3). For Lp(a), correlations were weak by both the UCSD and Roche assays, with all absolute r values ≤ 0.18 and all *P* values ≥ 0.14 for the UCSD assay and all absolute r values ≤ 0.11 and all *P* values ≥ 0.38 for the Roche assay. Similarly, OxPL-apoB and OxPL-apo(a) were not significantly correlated with any measured lipid variable, with only a modest nonsignificant inverse association observed between OxPL-apo(a) and apoB-48 (r = −0.23, *P* = 0.07).

In contrast, IgG MDA mimotope autoantibodies showed modest inverse correlations with LDL-C (r = −0.25, *P* = 0.04) and HDL-C (r = −0.26, *P* = 0.03), whereas no significant associations were observed with triglycerides, apoC-III, apoB, apoB-48, or chylomicron triglycerides. IgM MDA mimotope autoantibodies were likewise not significantly associated with most lipid variables, although borderline inverse associations were observed with apoC-III (r = −0.23, *P* = 0.06) and apoB-48 (r = −0.24, *P* = 0.05).

### Effect of olezarsen on Lp(a) and OxPL biomarkers

Despite producing the expected marked reductions in plasma triglycerides, olezarsen treatment did not significantly alter circulating Lp(a), OxPL-apoB, IgG MDA mimotope antibodies, or IgM MDA mimotope antibodies compared with placebo at either Day 169 or Day 365 (supplemental Table S4). A nominal increase in OxPL-apo(a) was observed with the 50-mg dose at Day 169 (*P* = 0.013), and a nominal difference in IgG MDA mimotope antibodies was observed with the 80-mg dose at Day 365 (*P* = 0.029); however, these isolated findings were not reproduced across doses or study visits and were considered unlikely to represent consistent treatment effects.

## Discussion

In this analysis of genetically confirmed FCS, severe disruption of LPL-dependent lipolysis in a chylomicron-dominant metabolic state was associated with markedly lower circulating Lp(a) concentrations despite similar apo(a) isoform characteristics. Across apo(a) isoform sizes, participants in Balance demonstrated a downward shift of the canonical inverse apo(a) isoform–Lp(a) relationship compared with the age- and sex-matched Essence-TIMI 73b cohort, which had moderate hypertriglyceridemia characterized predominantly by VLDL and remnant lipoproteins. The greatest separation was observed among individuals with smaller apo(a) isoforms. OxPL-apo(a) concentrations and the OxPL-apo(a)/OxPL-apoB ratio were also reduced, suggesting altered distribution of OxPL in the setting of markedly reduced Lp(a). Thus, the contrasting triglyceride phenotypes in Balance and Essence-TIMI 73b suggest that the nature and metabolic processing of triglyceride-rich lipoprotein particles may modify the phenotypic expression of genetically determined Lp(a) and its associated OxPL.

A central finding of this study is that the relationship between apo(a) isoform size and circulating Lp(a) concentration was preserved but shifted downward in FCS. This pattern was evident with both the Roche and UCSD isoform-independent assays, supporting the robustness of the observation across analytical platforms. Moreover, the similar distributions of predominant and minor apo(a) isoforms and their relative expression between Balance and Essence-TIMI 73b indicate that the lower Lp(a) concentrations in FCS cannot be explained by differences in apo(a) isoform distribution. These findings suggest that severe metabolic disturbances modify the phenotypic expression of genetically determined Lp(a) despite similar apo(a) isoform architecture.

The present findings extend the modest inverse association between plasma triglycerides and Lp(a) observed in population-based studies 5, 6, 7, 8 to the extreme metabolic setting of genetically confirmed FCS. Several mechanisms may contribute to the lower circulating Lp(a) concentrations observed in FCS, including altered apo(a)-apoB assembly, altered hepatic apo(a) secretion, or enhanced Lp(a) clearance associated with profound disturbances in triglyceride-rich lipoprotein metabolism. One possibility is reduced availability of suitable LDL-like particles for Lp(a) assembly. Although LDL-C was markedly lower in Balance, LDL-C reflects cholesterol mass rather than LDL particle number, and total apoB does not distinguish LDL-like particles from other apoB-containing lipoproteins. Thus, the present data cannot establish whether the number of suitable apoB-100-containing acceptor particles is reduced in FCS. Similarly, circulating free apo(a) was not measured, and we therefore cannot determine whether impaired assembly is accompanied by accumulation of unbound apo(a).

The comparison between Balance and Essence-TIMI 73b suggests that the metabolic relationship between TRLs and Lp(a) depends not simply on plasma triglyceride concentration, but on the class and metabolic fate of the particles carrying triglyceride. In Essence-TIMI 73b, moderate hypertriglyceridemia is characterized predominantly by VLDL and remnant particles that remain subject to LPL-mediated lipolysis and progressive intravascular remodeling. In this setting, our recent studies demonstrate marked triglyceride and cholesterol enrichment of apo(a)-containing particles, while apoC-III inhibition substantially remodels their lipid composition without reducing Lp(a) particle concentration. 23, 24 By contrast, FCS is characterized by profound impairment of LPL-dependent lipolysis and accumulation of large chylomicron-rich particles, with substantially reduced formation and remodeling of downstream remnant and LDL-like particles. In this setting, circulating Lp(a) was suppressed, particularly among individuals genetically predisposed to higher Lp(a) through small apo(a) isoforms. These contrasting phenotypes suggest that chylomicron-dominant and VLDL/remnant-rich triglyceride metabolism may influence Lp(a) differently and that the identity and intravascular processing of triglyceride-rich particles may be more important determinants of Lp(a) phenotype than triglyceride concentration itself.

The marked differences in triglyceride-rich lipoprotein particle biology between FCS and moderate hypertriglyceridemia may be mechanistically important. In VLDL/remnant-rich hypertriglyceridemia, ongoing LPL-dependent lipolysis generates progressively remodeled apoB-100-containing particles, whereas in FCS this pathway is profoundly impaired and the circulating triglyceride-rich lipoprotein pool is dominated by large chylomicron particles. Profound impairment of LPL-dependent lipolysis alters lipoprotein remodeling and could modify particle size, lipid composition, buoyancy, and apoB-100 conformation. Because Lp(a) assembly requires interaction of apo(a) with apoB-100 followed by covalent bond formation, altered structural properties of potential LDL-like acceptor particles could theoretically impair Lp(a) assembly even in the presence of an adequate number of apoB-containing particles. Direct measurements of LDL particle number and size, free apo(a), and the composition and structural properties of apoB-containing particles will be required to distinguish among these possibilities. Dedicated kinetic studies measuring apo(a) production, Lp(a) assembly, and Lp(a) clearance in FCS will ultimately be required to distinguish among these mechanisms.

Recent findings by Takov *et al.* (25) provide additional context for these observations. Using native Lp(a) immunocapture, proteomic, and lipidomic approaches, it was demonstrated that Lp(a) reproducibly associates with apoE and carries a defined triglyceride lipid signature, with Lp(a) particles being more buoyant than apoB-only particles. Moreover, the Lp(a)-associated lipidome was relatively stable postprandially, suggesting that triglyceride carriage by Lp(a) represents an intrinsic compositional feature rather than simply reflecting ambient plasma triglyceride concentrations. These observations reinforce the concept that Lp(a) composition is influenced by the lipoprotein environment in which the particle is assembled and remodeled rather than simply by ambient plasma triglyceride concentration. This distinction may be particularly relevant when comparing the VLDL/remnant-rich state of moderate hypertriglyceridemia with the chylomicron-dominant state of FCS, in which absent or markedly impaired LPL-dependent lipolysis profoundly alters the generation, composition, and remodeling of apoB-containing particles. Thus, the pool of apoB-containing particles available for apo(a) assembly may differ qualitatively as well as quantitatively, potentially influencing Lp(a) assembly, composition, stability, or clearance. Whether Lp(a) itself has altered triglyceride, apoE, or other lipid and protein composition in FCS cannot be determined from the present study and will require direct analysis of purified particles. Importantly, triglyceride enrichment of apo(a)-containing particles does not necessarily imply that intact Lp(a) shifts predominantly into the VLDL density range. In our complementary FPLC study of moderate hypertriglyceridemia, approximately 86%–87% of intact Lp(a) remained in non-TRL, predominantly LDL-sized fractions, whereas only approximately 13%–14% was detected in TRL fractions, despite substantial Lp(a)-associated triglyceride enrichment. (23) Thus, whether intact Lp(a) in FCS undergoes a more profound shift in size or density remains unknown and will require direct lipoprotein fractionation.

Our findings extend previous observations that Lp(a) concentrations are reduced in lipoprotein lipase deficiency and recent reports of lower Lp(a) levels in severe hypertriglyceridemia irrespective of genetic cause 10, 26. Unlike these earlier studies, the present analysis incorporated two standardized Lp(a) assays, detailed apo(a) isoform phenotyping, and measurements of oxidation-specific biomarkers, providing a more comprehensive characterization of the Lp(a) phenotype in FCS.

A second major finding relates to OxPL carriage in the setting of the altered Lp(a) phenotype. In normal human plasma, Lp(a) is the preferential carrier of E06-detectable OxPL among apoB-containing lipoproteins. In FCS, both OxPL-apo(a) and OxPL-apoB were reduced, but the reduction in OxPL-apo(a) was disproportionately greater, resulting in an approximately 70% lower OxPL-apo(a)/OxPL-apoB ratio. Thus, these data should not be interpreted as demonstrating absolute enrichment of non-Lp(a) apoB particles with OxPL, but rather as evidence for altered relative partitioning of OxPL away from Lp(a). Several mechanisms may contribute. The markedly reduced circulating Lp(a) particle concentration may decrease the available acceptor pool for OxPL, while absent LPL-dependent lipolysis may alter the surface composition, remodeling, residence time, and interparticle lipid exchange of apoB-containing lipoproteins. Altered composition of Lp(a) itself may also influence its capacity to acquire or retain OxPL. Notably, OxPL-apoB and OxPL-apo(a) were not significantly correlated with triglycerides, apoB-48, or chylomicron triglycerides within FCS, suggesting that the altered partitioning is not simply proportional to the contemporaneous burden of circulating triglyceride-rich particles. Because the present assays do not resolve the specific apoB-containing subclasses carrying OxPL, direct fractionation and compositional analysis will be required to determine whether OxPL is preferentially retained on particular non-Lp(a) particles in FCS.

Notably, the mean ratio observed in the Essence-TIMI 73b cohort is consistent with values previously reported in patients with established cardiovascular disease 22, 27. The OxPL-apo(a)/OxPL-apoB ratio showed only modest variation across apo(a) isoform categories in Essence-TIMI 73b, whereas FCS demonstrated a substantially stronger inverse trend with increasing apo(a) isoform size, consistent with altered relative carriage of OxPL among apoB-containing lipoproteins in the setting of functional deficiency of the LPL pathway. This effect was most evident among participants with progressively larger apo(a) isoforms, whereas the ratio remained relatively constant across comparable isoform categories in Essence-TIMI 73b. The absence of significant differences in IgG and IgM MDA mimotope autoantibodies further supports the interpretation that these findings reflect preferential carriage of OxPL rather than generalized oxidative stress.

Within the Balance cohort, Lp(a) showed little association with conventional lipid measures, including triglycerides, apoC-III, apoB, apoB-48, and chylomicron triglycerides. This lack of correlation despite the extreme triglyceride burden further suggests that plasma triglyceride concentration itself is not the principal determinant of the suppressed Lp(a) phenotype 28, 29. Rather, the phenotype appears to be associated with the underlying disturbance in triglyceride-rich lipoprotein metabolism—particularly the accumulation of chylomicrons and failure of normal LPL-dependent lipolysis, remnant generation, and apoB-particle remodeling. Together with the contrasting phenotype observed in the VLDL/remnant-rich Essence-TIMI 73b population, these findings support the concept that the class and metabolic processing of triglyceride-rich lipoproteins may differentially influence Lp(a) assembly, composition, stability, or clearance.

The lower Lp(a) phenotype may also have implications for cardiovascular risk in FCS 30, 31, 32. Although the relatively low ASCVD burden reported in genetically confirmed FCS is likely driven predominantly by lifelong low LDL-C and the accumulation of very large, relatively non-atherogenic triglyceride-rich particles, reduced lifelong exposure to Lp(a) and its associated OxPL may provide an additional contribution. Competition among lipoprotein particles for cellular uptake or hepatic clearance could represent another link between TRL metabolism and Lp(a), although the pathways responsible for Lp(a) clearance remain incompletely defined and this mechanism is speculative. Importantly, these observations should not be interpreted as evidence that elevated triglycerides or TRLs are generally protective, because remnant-rich hypertriglyceridemia outside FCS is associated with increased ASCVD risk.

This study has several limitations. First, this was a post hoc analysis and was not designed to directly investigate Lp(a) metabolism. Second, the sample size was limited by the rarity of genetically confirmed FCS, particularly within apo(a) isoform strata. Third, comparisons with Essence-TIMI 73b were cross-trial and based on age- and sex-matched participants with more modest hypertriglyceridemia; although apo(a) isoform characteristics were similar between cohorts, residual differences unrelated to FCS itself cannot be excluded.

In conclusion, genetically confirmed FCS is associated with a downward shift in circulating Lp(a) within apo(a) isoform strata, with the greatest differences among individuals with smaller apo(a) isoforms, despite preserved apo(a) isoform architecture. The accompanying reduction in the OxPL-apo(a)/OxPL-apoB ratio is consistent with altered relative partitioning of OxPL to non-Lp(a) apoB-containing lipoproteins in the setting of severe hypertriglyceridemia and lower Lp(a). In contrast to the triglyceride-enriched Lp(a) phenotype observed in VLDL/remnant-rich moderate hypertriglyceridemia, the chylomicron-dominant state of FCS is associated with suppression of circulating Lp(a). These findings suggest that the class and metabolic processing of triglyceride-rich lipoproteins, rather than triglyceride concentration alone, modify the phenotypic expression of genetically determined Lp(a) and its associated OxPL carriage.

## Data availability

Data requests from qualified researchers will be considered once all three of the following criteria are met: (1) 12 months from marketing approval of the study drug in both the United States and European Union; (2) 18 months from conclusion of the study; and (3) 6 months from publication of this article.

## Supplemental data

This article contains supplemental data.

## Conflict of interest

S. T. is a co-inventor and receives royalties from patents owned by University of California San Diego (UCSD), is a co-founder and has an equity interest in Oxitope, LLC, Kleanthi Diagnostics, LLC and Megaron Inc and has a dual appointment at UCSD and Ionis Pharmaceuticals. X. Y. has no conflicts. S. T., V. J. A., F. L., and E. W. are employees of Ionis Pharmaceuticals. S. M. M. is an employee of Medpace Reference Laboratories.
