# Supplementary material for: Deficiency of the lipoprotein lipase pathway modifies the phenotypic expression of genetically determined lipoprotein(a)
Source: J Lipid Res. 2026 Oct 5;67(11):101164. doi: 10.1016/j.jlr.2026.101164 (PMC13756623; doi:10.1016/j.jlr.2026.101164)
Supplement: Supplementary file 1 [file mmc1.docx]

**Supplementary Table 1**. Comparison of Balance UCSD versus ESSENCE Roche Lp(a) concentrations according to predominant apo(a) isoform size.

| **Predominant apo(a) isoform size (KIV repeats)** | **ESSENCE**  **N** | **ESSENCE**  **Lp(a) (Roche)**  **Mean (SD), nmol/L** | **Balance**  **N** | **Balance**  **Lp(a) (UCSD)**  **Mean (SD), nmol/L** | **% Lower Balance vs ESSENCE** | **Nominal**  **P Value** |  |
| --- | --- | --- | --- | --- | --- | --- | --- |
| <17 | 5 | 222.6 (124.0) | 7 | 31.3 (23.8) | 85.9 | 0.003 | |
| 17–20 | 16 | 144.5 (132.9) | 17 | 28.4 (49.7) | 80.4 | 0.004 | |
| 21–24 | 14 | 44.6 (56.7) | 10 | 4.35 (2.73) | 90.2 | 0.028 | |
| >24 | 31 | 9.46 (8.66) | 28 | 4.25 (3.72) | 55.1 | 0.001 | |

Predominant apo(a) isoform size was defined by the apo(a) isoform with the greater relative expression. ESSENCE Lp(a) was measured with the Roche Tina-quant Lipoprotein(a) Gen.2 assay. BALANCE Lp(a) was measured with the UCSD isoform-independent assay. Percent lower was calculated as 100 × (ESSENCE mean − FCS mean)/ESSENCE mean. P values compare participant-level ESSENCE Roche measurements with participant-level BALANCE Roche or UCSD measurements within each isoform category using two-sided Wilcoxon rank-sum tests. These cross-assay UCSD comparisons should be interpreted as supportive because ESSENCE samples were not measured with the UCSD assay. KIV indicates kringle IV; Lp(a), lipoprotein(a); and SD, standard deviation.
