# Supplementary material for: Deficiency of the lipoprotein lipase pathway modifies the phenotypic expression of genetically determined lipoprotein(a)
Source: J Lipid Res. 2026 Oct 5;67(11):101164. doi: 10.1016/j.jlr.2026.101164 (PMC13756623; doi:10.1016/j.jlr.2026.101164)
Supplement: Supplementary file 2 [file mmc2.docx]

**Supplemental Table 2.** Correlations among circulating Lp(a), oxidation-specific biomarkers, and MDA mimotope antibodies in the Balance familial chylomicronemia syndrome cohort.

| **Biomarker** | **Lp(a) (Roche)** | **OxPL-ApoB** | **OxPL-Apo(a)** | **IgG MDA Mimotope** | **IgM MDA Mimotope** |
| --- | --- | --- | --- | --- | --- |
| Lp(a) (UCSD) |  |  |  |  |  |
| r | 0.81 | 0.63 | 0.84 | 0.02 | 0.11 |
| P-value | <0.0001 | <0.0001 | <0.0001 | 0.87 | 0.37 |
| Lp(a) (Roche) |  |  |  |  |  |
| r |  | 0.55 | 0.81 | 0.05 | 0.05 |
| P-value |  | <0.0001 | <0.0001 | 0.67 | 0.72 |
| OxPL-ApoB |  |  |  |  |  |
| r |  |  | 0.71 | 0.03 | 0.17 |
| P-value |  |  | <0.0001 | 0.81 | 0.17 |
| OxPL-Apo(a) |  |  |  |  |  |
| r |  |  |  | 0.02 | 0.18 |
| P-value |  |  |  | 0.86 | 0.15 |
| IgG MDA mimotope |  |  |  |  |  |
| r |  |  |  |  | -0.07 |
| P-value |  |  |  |  | 0.60 |
|  |  |  |  |  |  |
