# Supplementary material for: Deficiency of the lipoprotein lipase pathway modifies the phenotypic expression of genetically determined lipoprotein(a)
Source: J Lipid Res. 2026 Oct 5;67(11):101164. doi: 10.1016/j.jlr.2026.101164 (PMC13756623; doi:10.1016/j.jlr.2026.101164)
Supplement: Supplementary file 3 [file mmc3.docx]

**Supplemental Table 3.** Correlations of Lp(a), OxPL-Related Biomarkers, and MDA Autoantibodies with Lipid variables in the Baseline Study.

| **Biomarker** | **TG** | **ApoC-III** | **LDL-C** | **HDL-C** | **ApoB** | **ApoB48** | **Chylomicron TG** |
| --- | --- | --- | --- | --- | --- | --- | --- |
| Lp(a) (UCSD) |  |  |  |  |  |  |  |
| r | -0.12 | -0.04 | 0.08 | 0.02 | 0.03 | -0.18 | -0.10 |
| P-value | 0.34 | 0.76 | 0.53 | 0.86 | 0.78 | 0.14 | 0.42 |
| Lp(a) (Roche) |  |  |  |  |  |  |  |
| r | 0.06 | -0.06 | -0.11 | -0.08 | -0.06 | -0.08 | 0.02 |
| P-value | 0.66 | 0.64 | 0.38 | 0.38 | 0.65 | 0.53 | 0.89 |
| OxPL-apoB |  |  |  |  |  |  |  |
| r | -0.07 | -0.01 | -0.08 | 0.00 | -0.14 | -0.18 | -0.07 |
| P-value | 0.55 | 0.92 | 0.51 | 1.00 | 0.25 | 0.14 | 0.60 |
| OxPL-apo(a) |  |  |  |  |  |  |  |
| r | -0.06 | -0.05 | 0.01 | 0.07 | -0.03 | -0.23 | -0.04 |
| P-value | 0.65 | 0.68 | 0.96 | 0.57 | 0.82 | 0.07 | 0.78 |
| IgG MDA mimotope |  |  |  |  |  |  |  |
| r | -0.01 | -0.091 | -0.25 | -0.26 | -0.17 | -0.03 | -0.00 |
| P-value | 0.96 | 0.46 | 0.04 | 0.03 | 0.18 | 0.81 | 0.98 |
| IgM MDA mimotope |  |  |  |  |  |  |  |
| r | 0.03 | -0.23 | -0.00 | -0.12 | 0.01 | -0.24 | 0.08 |
| P-value | 0.79 | 0.06 | 1.00 | 0.33 | 0.94 | 0.05 | 0.54 |
