# Supplementary material for: Deficiency of the lipoprotein lipase pathway modifies the phenotypic expression of genetically determined lipoprotein(a)
Source: J Lipid Res. 2026 Oct 5;67(11):101164. doi: 10.1016/j.jlr.2026.101164 (PMC13756623; doi:10.1016/j.jlr.2026.101164)
Supplement: Supplementary file 4 [file mmc4.docx]

| **Supplementary Table 4**. Median concentrations of Lp(a), OxPL-related biomarkers, and anti-MDA antibodies at Days 169 and 365 by treatment group. | | | |
| --- | --- | --- | --- |
|  |  |  |  |
| **Variable** | **Placebo** | **Olezarsen 50 mg** | **Olezarsen 80 mg** |
|  | N=23 | N=21 | N=22 |
| Baseline Triglycerides, Median (IQR) | 2493 (1653,3605) | 2678 (1743.5,3168) | 2086 (1778,3141) |
| Day 169 Triglycerides Median (IQR) | 2225 (1486,3456) | 2152 (1524.4,2678) | 1414 1049,1954) |
| Mean % change from baseline to Day 169 | 8.7 | -14.5 | -38.6 |
| P-Value Day 169 (olezarsen vs. placebo) |  | 0.1016 | 0.0012 |
| Day 365 Triglycerides Median (IQR) | 2929 (1734,3786) | 2002(1078,2939) | 1206(1004,2039) |
| Mean % change from baseline to Day 365 | 20.6 | -25.5 | -40.9 |
| P-Value Day 365 (olezarsen vs. placebo) |  | 0.0057 | 0.0010 |
|  |  |  |  |
| Baseline Total cholesterol, Mean (SD) | 286 (113.9) | 323 (100.5) | 277.4(99.27) |
| Day 169 Total cholesterol, Mean (SD) | 251 (205,394) | 270 (196,309) | 188 (178,258) |
| Mean % change from baseline to Day 169 | 3.9 | -15.6 | -18.3 |
| P-Value Day 169 (olezarsen vs. placebo) |  | 0.0440 | 0.0036 |
| Day 365 Total cholesterol, Mean (SD) | 295 (249,351) | 269 (197,325) | 195 (141,232) |
| Mean % change from baseline to Day 365 | 11.8 | -19.1 | -24.1 |
| P-Value Day 365 (olezarsen vs. placebo) |  | 0.0071 | 0.0016 |
|  |  |  |  |
| Baseline Lp(a) (Roche), Median (IQR) | 8.25 (4.25-45.7) | 6.80 (4.50-40.90) | 12.9 (4.80-29.7) |
| Day 169 Lp(a) Median (IQR) | 9.90 (3.20-20.2) | 11.0 (5.20-41.0) | 13.2 (4.75-33.3) |
| P-Value Day 169 (olezarsen vs. placebo) |  | 0.18 | 0.79 |
| Day 365 Lp(a) Median (IQR) | 8.10 (3.9-63.3) | 6.95 (5.05-52.2) | 14.3 (5.70-24.0) |
| P-Value Day 365 (olezarsen vs. placebo) |  | 0.56 | 0.48 |
|  |  |  |  |
| Baseline Lp(a) (UCSD), Median (IQR) | 3.43 (0.99-16.4) | 3.06 (2.58-15.7) | 6.06 (3.96-8.00) |
| Day 169 Lp(a), Median (IQR) | 1.80 (1.12-10.1) | 2.78 (1.22-17.6) | 4.94 (1.70-14.4) |
| P-Value Day 169 (olezarsen vs. placebo) |  | 0.87 | 0.99 |
| Day 365 Lp(a), Median (IQR) | 2.07 (0.89-18.9) | 13.9 (2.38-19.5) | 3.31 (1.42-6.99) |
| P-Value Day 365 (olezarsen vs. placebo) |  | 0.73 | 0.25 |
|  |  |  |  |
| Baseline OxPL-apoB, Median (IQR) | 4.93 (3.62-11.3) | 4.15 (3.83-7.10) | 5.42 (4.34-6.55) |
| Day 169 OxPL-apoB, Median (IQR) | 4.48 (3.07-6.03) | 4.07 (2.95-5.57) | 4.77 (3.01-6.46) |
| P-Value Day 169 (olezarsen vs. placebo) |  | 0.77 | 0.95 |
| Day 365 OxPL-apoB, Median (IQR) | 3.87 (3.31-5.42) | 4.55 (3.12-5.71) | 4.19 (2.63-5.86) |
| P-Value Day 365 (olezarsen vs. placebo) |  | 0.75 | 0.56 |
|  |  |  |  |
| Baseline OxPL-apo(a), Median (IQR) | 2.43 (0.70-14.9) | 2.18 (0.90-17.4) | 3.53 (1.90-9.36) |
| Day 169 OxPL-apo(a), Median (IQR) | 0.78 (0.49-4.66) | 2.73 (0.95-16.0) | 2.07 (1.18-13.5) |
| P-Value Day 169 (olezarsen vs. placebo) |  | 0.013 | 0.108 |
| Day 365 OxPL-apo(a), Median (IQR) | 1.79 (0.60-19.6) | 1.95 (0.66-19.2) | 2.25 (0.66-6.47) |
| P-Value Day 365 (olezarsen vs. placebo) |  | 0.72 | 0.29 |
|  |  |  |  |
| Baseline IgM MDA mimotope, Median (IQR) | 4890 (3474-6823) | 5027 (3957-6662) | 4865 (3340-7370) |
| Day 169 IgM MDA mimotope, Median (IQR) | 4361 (3284-5176) | 4988 (3567-8608) | 5458 (4146-7406) |
| P-Value Day 169 (olezarsen vs. placebo) |  | 0.15 | 0.15 |
| Day 365 IgM MDA mimotope, Median (IQR) | 4453 (3525-5084) | 5130 (3991-6762) | 4626 (3911-6162) |
| P-Value Day 365 (olezarsen vs. placebo) |  | 0.31 | 0.87 |
|  |  |  |  |
| Baseline IgG MDA mimotope, Median (IQR) | 1839 (1074-2468) | 1473 (1105-2119) | 1237 (1009-1774) |
| Day 169 IgG MDA mimotope, Median (IQR) | 1677 (1051-2306) | 1397 (1120-1636) | 1494 (1155-1830) |
| P-Value Day 169 (olezarsen vs. placebo) |  | 0.53 | 0.14 |
| Day 365 IgG MDA mimotope, Median (IQR) | 1735 (952-2979) | 1526 (1300-1726) | 1509 (1116-2614) |
| P-Value Day 365 (olezarsen vs. placebo) |  | 0.71 | 0.029 |
